# Grains in a Modern Time: A Comprehensive Review of Compositions and Understanding Their Role in Type 2 Diabetes and Cancer

**DOI:** 10.3390/foods13132112

**Published:** 2024-07-02

**Authors:** Jabir Khan, Palwasha Gul, Kunlun Liu

**Affiliations:** 1 College of Food Science and Engineering, Henan University of Technology, Zhengzhou 450001, China; jabirkhan@163.com (J.K.); palwasha.gull68@outlook.com (P.G.); 2 School of Food and Strategic Reserves, Henan University of Technology, Zhengzhou 450001, China

**Keywords:** pseudo cereals, cereals, protein, dietary fibers, phenolic acids, T2D, cancer

## Abstract

Globally, type 2 diabetes (T2D) and Cancer are the major causes of morbidity and mortality worldwide and are considered to be two of the most significant public health concerns of the 21st century. Over the next two decades, the global burden is expected to increase by approximately 60%. Several observational studies as well as clinical trials have demonstrated the health benefits of consuming whole grains to lower the risk of several chronic non-communicable diseases including T2D and cancer. Cereals grains are the primary source of energy in the human diet. The most widely consumed pseudo cereals include (quinoa, amaranth, and buckwheat) and cereals (wheat, rice, and corn). From a nutritional perspective, both pseudo cereals and cereals are recognized for their complete protein, essential amino acids, dietary fibers, and phenolic acids. The bran layer of the seed contains the majority of these components. Greater intake of whole grains rather than refined grains has been consistently linked to a lower risk of T2D and cancer. Due to their superior nutritional compositions, whole grains make them a preferred choice over refined grains. The modulatory effects of whole grains on T2D and cancer are also likely to be influenced by several mechanisms; some of these effects may be direct while others involve altering the composition of gut microbiota, increasing the abundance of beneficial bacteria, and lowering harmful bacteria, increasing insulin sensitivity, lowering solubility of free bile acids, breaking protein down into peptides and amino acids, producing short-chain fatty acids (SCFAs), and other beneficial metabolites that promote the proliferation in the colon which modulate the antidiabetic and anticancer pathway. Thus, the present review had two aims. First, it summarized the recent knowledge about the nutritional composition and bioactive acids in pseudo cereals (quinoa, amaranth, and buckwheat) and cereals (wheat, rice, and corn); the second section summarized and discussed the progress in recent human studies, such as observational (cross-sectional studies, case-control studies, and cohort studies) and intervention studies to understand their role in T2D and cancer including the potential mechanism. Overall, according to the scientific data, whole grain consumption may reduce the incidence of T2D and cancer. Future studies should carry out randomized controlled trials to validate observational results and establish causality. In addition, the current manuscript encourages researchers to investigate the specific mechanisms by which whole grains exert their beneficial effects on health by examining the effects of different types of specific protein, dietary fibers, and phenolic acids that might help to prevent or treat T2D and cancer.

## 1. Introduction

Globally, type 2 diabetes (T2D) and cancer are the leading causes of morbidity and mortality and are considered as two of the most serious public health concerns of the 21st century. According to the International Diabetes Federation (IDF) 2021 report, an estimated 537 million adults worldwide have diabetes, with 783 million expected to have the condition by 2045 [1]. Additionally, according to World Health Organization report (WHO) reports, every year 1.5 million deaths are directly attributed to diabetes worldwide in low- and middle-income countries [2]. The WHO 2023 report on cancer estimated that there were 10 million cancer related deaths and 20 million additional cases of cancer worldwide. Globally, it is expected in 2040 that there will be over 30 million more cases of cancer worldwide, with the majority of cases in low- and middle-income countries [3]. Over the past several years, there has been a gradual increase in the number of cases and incidences of T2D and cancer. The increasing incidences of T2D and cancer worldwide represent a major risk to public health in underdeveloped as well as developing countries. The American Institute for Cancer Research and the U.S. Department of Health and Human Services declared, “whole grains as an important part of a healthy diet” [4,5]. Several epidemiological studies have given convincing results for the significant role of the whole grain diet in lowering the incidence of T2D [6,7] and cancer [8,9,10]. In contrast, some studies on refined grains have found a negative relationship between high refined grain consumption and an increased risk of T2D [11,12,13] and cancer [14]. These findings support the health recommendations to replace refined grains with whole grains. According to the American Association of Cereal Chemists International, whole grains are composed of intact ground, cracked, or flaked kernels that contain a starchy endosperm, germ, and bran [15]. In contrast, refined grain products do not contain one or more integral kernel components.

Whole grains provide protein, dietary fiber, and phenolic acids, all of which are helpful to one’s health [16,17,18,19]. Consuming whole grain protein can alter the composition of the gut microbiota enhancing the helpful bacteria and suppressing harmful bacteria [20]. The stomach and small intestine digest whole grain protein, converting it into peptides and amino acids [20]. Amino acids are absorbed into the bloodstream and carried to the large intestine colon [21]. The gut microbiota in the colon ferments the amino acids, producing short-chain fatty acids (SCFAs), and other metabolites that stimulate the proliferation of beneficial bacteria in the colon and also play an essential role in the reduction of T2D and cancer [18,22,23]. Furthermore, the bacterial gut population digests dietary fibers and produces SCFAs [24], which modulate gut hormone production and influence glucose and lipid metabolism which may reduce the risk of type 2 diabetes [24,25]. Furthermore, dietary fiber can remove nitrite from the stomach and reduce the amounts of nitros compounds under high acid conditions, because nitrates enhance the risk of stomach cancer [26,27]. It has been demonstrated that phenolic acids and dietary fibers included in whole grains have a substantial impact on human health and protect against chronic non-communicable diseases [28,29].

A comprehensive systematic review of 11 cohort studies published in 2022 by Ghanbari-Gohari et al., [7], discovered that whole grain consumption is related to a lower incidence of T2D in the follow up of 463,282 individuals. The study found that intake of 50 g of whole grains was related to a 23% decreased incidence of T2D. The author indicated that increasing whole grain consumption might be more useful in lowering the risk of T2D. More recently, in 2024 a comprehensive review and meta-analysis of prospective trials in two prospective cohorts by Watling et al. [8] explored the influence of whole grains on cancer and revealed that 16 g/day of whole grains can reduce the risk of cancer. This study comprised 111,396 people from the prostate, lung, colorectal, and ovarian cancers and discovered that dietary fiber and whole grains were inversely related to liver cancer risk. The results of this study showed that consuming 10 g of dietary fiber and 16 g of whole grains per day has been associated with a decreased risk of cancer.

Consumption of whole grain products, which have much higher protein, dietary fiber, and phenolic contents than refined grains, and have a dietary fiber profile with a good balance of soluble and insoluble fibers, can surely make a significant impact on reducing the risk of diseases. However, nutritional approaches that emphasize grain intake are limited, particularly for whole grains [30,31,32]. While currently published recommendations are only available to the general population and are not influenced by the risk level of individual health outcomes [4,33], particular guidance for T2D and cancer prevention are urgently required.

Thus, the purpose of this review was to first discuss the nutritional compositions of pseudo cereals (quinoa, amaranth, and buckwheat) and cereals (wheat, rice, and corn), as well as the essential and non-essential amino acids, dietary fibers (total, insoluble, and soluble), phenolic acids (hydroxycinnamic acids and hydroxybenzoic acids) and their antioxidant activates. Secondly, we studied the relationship between whole grain intake and the incidence of T2D and cancer from human studies, including observational and intervention studies, as well as the possible mechanism behind the intake of whole grains and T2D and cancer. Recent research indicates that whole grains including protein, dietary fiber, and phenolic acids may be more beneficial than individual isolated components. Future research should include more clinical trials and epidemiological and randomized controlled trials to validate observational results. Additionally, to investigate the specific mechanisms by which whole grains exert their beneficial effects on health by examining the effects of specific proteins, dietary fibers, and phenolic acids that might help to prevent or treat T2D and cancer.

## 2. Material and Methods

**Figure 1:**
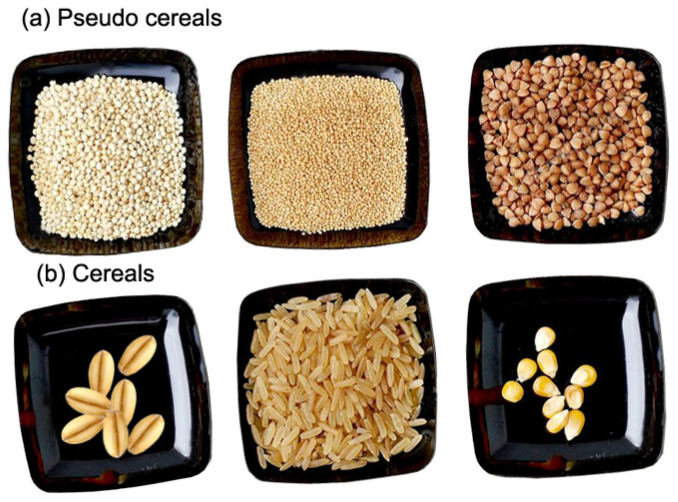
Images of whole grains included in the study: (**a**) pseudo cereals, quinoa, amaranth, and buckwheat; (**b**) cereals, wheat, rice, and corn.

We have reviewed and summarized the nutritional characteristics of three targeted pseudo cereals (quinoa, amaranth, and buckwheat) and three cereals (wheat, rice, and corn) (Figure 1), including their moisture %wet basis (w.b), fat (%), ash (%), carbohydrate (%), crude protein (%), amino acid composition (g/100 g), and phenolic acids (µg/g) as well as their antioxidant activates. Following that, we reviewed and discussed the recent findings concerning the link between whole grain consumption and chronic non-communicable diseases like T2D and cancer. Then we reviewed the current research progress on the relationship between whole grain consumption and chronic non-communicable diseases like T2D and Cancer. All of the materials for this review were sourced from PubMed and Google Scholar, including human studies such as observational (cross-sectional, case-control, and cohort studies) and interventional research. In the current review, relevant data published in English in peer-reviewed journals were included for discussion. However, any information available in the form of conference abstracts, books, and unpublished studies was eliminated.

## 3. Nutritional Composition of Pseudo Cereals and Cereals

### 3.1. Fat in Pseudo Cereals and Cereals

In whole grain compositions, amaranth contains the highest fat content (6.4 to 8.0%) [34,35,36] among all other grains followed by quinoa (4.9 to 7.5%) [37,38], buckwheat (1.4 to 7.4%) [39,40], corn (3.8–4.7%) [41,42], wheat (1.7–3.3%) [43,44,45,46], and rice (1.5–2.2%) [47,48] (Table 1). The highest fat level in pseudo cereal is due to a high concentration of unsaturated fatty acids, including linolenic acid. Among cereal grains, corn contains the highest content of fat (3.8–4.7%) [41], which has primarily mono (30%) and poly-unsaturated (50%) lipids, with a small amount of saturated lipids (20%) [41,42]. Furthermore, fat is the second most important component of amaranth grain, with a larger concentration than other cereals. Fat is characterized by the high concentration of unsaturated fatty acids as well as saturated fatty acids. Linoleic acid is a major fatty acid in oil, making up more than half of the content of amaranth grain seeds [36]. The next highest is oleic acid (more than 20%), followed by palmitic acid (almost 20%) [49]. Amaranth contains two to three times the fat content of quinoa, buckwheat, and all other cereal grains, indicating how widely different grains are from one another.

### 3.2. Carbohydrates and Starch in Pseudo Cereals and Cereals

Carbohydrates are the primary nutritious components of pseudo cereal grains, accounting for 60 to 80% of the seed dry weight (d.w) [50,51,52]. Carbohydrates are essential in nutrition and affect various metabolic processes [25,53]. Quinoa, amaranth, and buckwheat, like other cereals, contain high levels of carbohydrates (50–75%), which are mostly composed of starch and non-starchy polysaccharides. According to the Table 1, we found that wheat contains the highest content of carbohydrate: wheat (61.6–83.1%) [45,46,54,55,56,57,58,59], buckwheat (63.1–82.1%) [50,51,52,60,61,62], rice (71.1–78.2%) [47,48,62] quinoa (48.5–77.0%) [35,38,60,61], corn (65.0–74.3%) [33,41,42], and amaranth (63.1–70.0%) [34,35,36,50]. Starch is the primary carbohydrate, accounting for 58.1–64.2%, 65.0–75.0%, and 54.5–57.4% of total seed dry weight in quinoa, amaranth, and buckwheat, respectively [62]. Buckwheat starch contains more amylose (18.3–47% of total starch) than quinoa (11–12%) and amaranth (7.8–34.3%) [62]. Starch in the endosperm is a key component of rice grain, accounting for 50% to 90% of its dry weight [47,48]. The chemical composition of amaranth seeds is dominated by carbohydrates, with starch accounting for 50 to 60% of the overall mass of the seeds, or more than 90% of the carbohydrates present. Starch granules, which are found mostly in the endosperm, have a polygonal shape and a strong swelling capacity [63].

### 3.3. Protein in Pseudo Cereals and Cereals

The protein content and profile of all plants are determined by genotype and growing conditions. Compared to cereals, pseudo cereals offer superior nutritional value, primarily due to their higher protein levels, which vary from 9.1 to 16.7% for quinoa [35,38,60], 13.1–21.5% for amaranth [34,35,36,50], and 5.7–14.2% for buckwheat [50,51,52,60,61,62]; cereals such as wheat have 8–19.0% [45,46,54,55,56,57,58,59], 6.8–7.3% for rice [47,48,62], and 8.8–9.4% for corn [41,42] Table 1.

The major protein fractions found in quinoa grains are 11S-type globulins and 2S albumins, which account for 27.9 to 60.2% and 13.2 to 42.3% of total seed proteins, respectively, followed by glutelins (18.1–31.6); prolamins comprise a lower protein proportion (0.5–19.3% of total seed protein) [63,64]. Amaranthine is an essential protein found in the globulins portion with a molecular weight of more than 300kDa and three subunits: proamaranthine, one basic peptide, and one acidic [35]. Buckwheat seeds include primarily 8S and 13S globulin proteins. Furthermore, 2S globulins, glutelins, and prolamins are minor protein components of common wheat and buckwheat [22].

Quinoa is an excellent source of protein [65]. According to Abugoch’s reports [66], the proteins found in quinoa grains include albumins (35%) and globulins (37%), with a smaller proportion of prolamins. Quinoa proteins are of equal grade to milk protein (casein) and provide all essential amino acids [66,67], thus it is considered a complete diet [68]. Electrophoretic investigations revealed that quinoa proteins are composed of two major fractions: 11S-globulin and 2S-protein; 11S-globulin, also known as chenopodium [69], comprises nearly 37 percent of the total protein. This fraction consists of polypeptides with molecular weights of (22 to 23 and 32 to 39 kDa), as well as a relatively low concentration of amino acids (methionine and cysteine). The 2S-protein fraction has a molecular weight of 9 kDa and is rich in cysteine, arginine, and histidine, but low in methionine [69]. Proteins from amaranth seeds are rich in essential amino acids and have a high digestibility of around 90% [70,71,72,73]. Three primary proteins—albumin (40%), globulin (20%), and glutelin (25–30%)—as well as a little amount of prolamine (2–4%) are present in amaranth grains [74]. As a result of their inability to produce gluten, amaranth flour is recommended for those with celiac disease. Furthermore, amaranth contains lysine, which is a limit amino acid in cereal grains like wheat and others ranging from 363 to 421 mg/g N; the same quantity is found in soy [71]. Additionally, compared to basic legumes (1.4% on average), amaranth proteins have a significant number of amino acids containing sulfur (2–5%), methionine, cystine, and cysteine [55,75,76].

**Table 1:** Nutritional composition of pseudo cereals (quinoa, amaranth, and buckwheat); and cereals (wheat, rice, and corn) grains in percentage (%).

Pseudo Cereals							
Whole Grains	Moisture (w.b)	Fat	Ash	Carbohydrates	Starch	Crude Protein	References
Quinoa	8.2–13.1	4.9–7.5	4.1	48.5–77.0	58.1–64.2	9.1–16.7	[35,37,38,60,62,76]
Amaranth	8.9–9.4	6.4–8.0	3.3	63.1–70.0	65.0–75.0	13.1–21.5	[34,35,36,50,60,77]
Buckwheat	10–11	1.4–7.4	1.33–3.09	62.1–82.1	54.5–57.4	5.7–14.2	[35,39,40,51,52,60,78]
Cereals							
Wheat	12–13	1.72–3.3	1.7–1.9	62.6–83.1	60–75	8–19.0	[43,45,46,54,55,56,57,58,59]
Rice	5.0–12.7	1.5–2.2	0.5–3.5	71.1–78.2	50–90	6.8–7.3	[47,48,62]
Corn	10.4	3.8–4.7	1.33–1.44	65.0–74.3	72.8	8.8–9.4	[41,42]

It is known that buckwheat is an excellent source of lipids, antioxidants, organic acids, dietary fiber, phenolic acid, and high biological value proteins that do not form gluten and a balanced amino acid composition (high quantities of lysine and arginine, compared to cereals). Moreover, buckwheat seeds are mostly composed of two types of proteins: 8S and 13S globulins, but also contain small amounts of 2S globulins, glutelins, and prolamins [79]. Buckwheat contains 5–14% protein, of which 35% is found in the endosperm, 55% in the embryo, and the remaining portion is found in the shell [80,81]. On the other hand, cereal proteins contain 80–90% protein in the endosperm and 10–15% of the embryo [82].

Globally, rice as is the primary source of nutrition for the majority of people. According to Hirawan et al. [47], 100 g of brown rice contains about 7.3 g of protein, 2.2 g of fat, and 71.1 g of carbohydrates. Furthermore, rice bran contains (10–15%) protein, making it superior among other cereal grains due to its unique allergenic and anticancer attributes [83]. Similarly, whole corn possesses 8.8% protein, 3.8% fat, 1.33% ash, and 65.0% carbs; whole wheat contains 12.7% protein, 1.6% ash, 1.72% fat, and 61.6% carbs in 100 g of whole wheat [43].

Table 1 represents the nutritional composition of pseudo cereal and cereals and the data indicates that buckwheat carbohydrate content ranges from 62.6 to 82.1% which is very close to that of wheat which ranges from 62.1 to 83.1%. According to the literature, wheat has the highest carbohydrate content while amaranth has the lowest, followed by buckwheat, rice, quinoa, and corn. In terms of protein content, amaranth has the highest percentage (13.1–21.5%), while rice has the lowest (6.8–7.3%). The descending order of protein content of whole grains cereals and pseudo cereals is amaranth, wheat, quinoa, buckwheat corn, and rice, indicating that pseudo cereals have the highest protein content when compared to cereals. Furthermore, rice has the highest starch content among all other cereals grains, while buckwheat has the lowest; both wheat and amaranth have values of 60 to 75% and 65.0 to 75.0% which are closely related to each other, respectively. Pseudo cereals are a popular trend in human diets because they are gluten-free (GF) and have high nutritional and nutraceutical value. Furthermore, consumption of these pseudo cereals plays an essential role in lowering the risk of numerous chronic non-communicable diseases, such as T2D [6,7,24,25] and cancer [8,9,10,26,27]. In addition, a recent study has highlighted the potential health benefits of pseudo cereals, presenting these crops as valuable resources for functional food creation [50,51]. However, more experiments are needed to confirm the carbohydrate and protein contents of pseudo cereals and cereals.

### 3.4. Amino Acids in Pseudo Cereals and Cereals

Pseudo cereals have a balanced amino acid composition with greater amounts of lysine and arginine compared to cereal grains, and have been recognized as a good source of high biological value proteins that do not produce gluten [84,85,86,87]. As previously discussed in the nutritional composition section of pseudo cereals and cereals, several studies have confirmed that quinoa contains high-quality protein and its classification as gluten-free plays a key role in celiac diseases [68]. Furthermore, quinoa is a complete protein containing all of the necessary amino acids which are required for human growth and development and has a protein efficiency ratio comparable to milk casein [88]. Quinoa’s main protein components are globulins and albumins [64,68]. Quinoa protein 2S-type albumin has a high cysteine, arginine, and histidine content [89]. Quinoa contains the highest amount of Leucine (2.3–9.4 g/100 g), followed by methionine (0.3–9.1 g/100 g) protein, and the lowest concentration of tryptophan (0.6–1.9 g/100 g) protein in essential amino acids [61], while in non-essential amino acids, quinoa contains the highest concentration of arginine (6.9–13.6 g/100 g) and the lowest concentration of cysteine (0.1–2.7 g/100 g), respectively [61] (Table 2). Amaranth seed proteins are rich in essential amino acids with a high digestibility of around 90% [70,71,72]. Amaranth contains the highest content of lysine (4.8–8.00g/100 g) followed by leucine (4.2–6.9 g/100 g) protein being lowest in tryptophan (0.9–1.8 g/100 g) in essential amino acids [50], while in non-essential amino acids, amaranth contains the highest content of glutamate (14.4–17.7 g/100 g) and the lowest amount of cysteine (2.1–3.6 g/100 g), respectively [36,55,63]. Furthermore, as discussed in the previous section, the major protein in buckwheat is 13S globulin, which is regarded as a rare vegetable protein with blood cholesterol-lowering properties. Buckwheat also includes lectins, which help to reduce the proliferation of both spontaneous and induced tumors [90]. Buckwheat has a balanced amino acid content, with a significant amount of lysine (4.2–8.6 g/100 g) and low levels of tryptophan (0.9–1.8 g/100 g) among essential amino acids [52,62] It also contains the highest amount of glutamate (23.2–24.4 g/100 g) and the lowest amount of cysteine (0.8–3.5 g/100 g) among non-essential amino acids [52,61].

Wheat contains the highest amount of leucine (4.1–6.3 g/100 g) and the lowest amount of tryptophan (0.7–1.2 g/100 g) among essential amino acids [49,91,92], while it contains the highest amount of serine (6.1–6.7 g/100 g) and the lowest amount of proline (1.5–2.3 g/100 g) among non-essential amino acids [92,93,94,95]. Rice contains the highest amount of phenylalanine (5.2–9.1 g/100 g), followed by leucine (8.2–8.9 g/100 g) protein, and the lowest amount of tryptophan (1.0–1.5 g/100 g) in essential amino acids [49,91,96], while also having the highest level of glutamate (7.2–20.8 g/100 g) and the lowest level of cysteine (1.6–2.0 g/100 g) in non-essential amino acids [93,96]. Furthermore, corn contains the highest content of phenylalanine (4.8–10.6 g/100 g), followed by valine (3.6–5.1 g/100 g) protein, and the lowest content of methionine (0.6–0.7 g/100 g) in essential amino acids [49,91,93,96]; it also has the highest content of glutamate (7.13–15.8 g/100 g) and the lowest content of cysteine (2.1–2.3 g/100 g), respectively [93,96,97].

**Table 2:** Essential and non-essential amino acids in pseudo cereals and cereals.

Essential Amino Acids										
Pseudo Cereals										
Whole Grains	Th	Va	Ph	Is	Le	Me	Tr	Ly	Hi	References
Quinoa	2.1–8.9	0.8–6.1	3.0–4.7	0.8–7.4	2.3–9.4	0.3–9.1	0.6–1.9	2.4–7.8	1.4–5.4	[61]
Amaranth	3.3–5.0	3.9–5.0	3.7–4.7	2.7–4.2	4.2–6.9	1.6–4.6	0.9–1.8	4.8–8.0	1.9–3.8	[36,50]
Buckwheat	3.9–4.0	2.3–6.1	1.3–7.2	1.1–4.1	2.2–7.6	0.5–2.5	0.7–1.8	4.2–8.6	1.8–4.9	[52,63]
Cereals										
Wheat	1.8–2.7	2.4–4.1	2.8–8.1	2.2–4.1	4.1–6.3	0.9–1.2	0.7–1.2	1.7–2.6	0.2- 1.3	[49,91,92]
Rice	3.2–3.7	4.5–4.5	5.2–9.1	2.8–4.5	8.2–8.9	1.0–1.6	1.0–1.5	3.3–3.8	0.1–1.7	[49,91,96]
Corn	1.1–4.0	3.6–5.1	4.8–10.6	2.3–4.6	1.3–3.8	0.6–0.7	0.6- 1.1	2.6–1.9	2.3- 2.6	[49,91,93,96]
Non-Essential Amino Acids										
Pseudo cereals										
**Whole Grains**	**As**	**Glu**	**Se**	**Gly**	**Ar**	**Al**	**Ty**	**Cy**	**Pr**	**References**
Quinoa	8.0	13.2	3.4–5.7	2.2–6.1	6.9–13.6	3.2–5.7	2.5–3.7	0.1–2.7	2.3–5.5	[61,64]
Amaranth	7.3–10.7	14.4–17.7	4.9–9.3	6.7–15.2	8.7–15.6	3.5–6.2	3.3–3.7	2.1–3.6	2.82–4.6	[33,55]
Buckwheat	7.6–16.6	23.2–24.4	3.2–8.6	6.2–13.2	10.5–11.3	4.6–9.6	0.6–4.9	0.8–3.5	2.6–8.8	[52,62]
Cereals										
Wheat	4.2–6.6	2.8–3.5	6.1–6.9	4.6–6.31	4.7–7.2	3.8–5.4	1.8–3.8	1.4–3.0	1.5–2.3	[92,93,94,95]
Rice	4.2–10.7	7.2–20.8	4.0–5.7	3.9–5.2	7.2–8.2	4.5–6.3	2.3–3.2	1.6–2.0	4.8–5.4	[93,96]
Corn	4.7–6.0	7.13–15.8	5.0–6.4	2.5–4.0	4.3–10.3	5.1–7.9	3.0–4.8	2.1–2.3	1.1–2.8	[93,96,98]

Table 2: Amino acid compositions of pseudo cereals and cereals; T; Threonine, V; Valine, P; Phenylalanine, I; Isoleucine, L; Leucine, M; Methionine, T; Tryptophan, L; Lysine, A; Aspartate, G; Glutamate, S; Serine, H; Histidine, G; Glycine, A; Arginine, A; Alanine, T; Tyrosine, C; Cysteine, P; Proline.

Table 2 reviews nine essential amino acids (g/100 g protein) and nine non-essential amino acids (g/100 g protein) in the pseudo cereals quinoa, amaranth, and buckwheat, and in the cereals wheat, rice, and corn. In essential amino acids, except for quinoa, amaranth and buckwheat contain the highest content of lysine, while this content is ranked fourth in quinoa. Furthermore, lysine is most prevalent in pseudo cereal grains, and phenylalanine is most prevalent in cereal grains. Most interestingly, the lysine content of all pseudo cereal grains is closely related to each other, whereas cereal grains are lower in lysine. Methionine is second in quinoa, leucine in amaranth, while leucine is ranked second in wheat and rice, and valine is second in corn. In terms of non-essential amino acids, quinoa and wheat contain the most arginine, while amaranth, buckwheat, rice, and corn contain the most glutamate. Except for wheat, all other grains contain a small amount of cysteine, which is ranked second in wheat, and the ranking becomes more variable in the rest of the essential amino acids found in pseudo cereals and cereals. Corn, quinoa, rice, buckwheat, wheat, and amaranth are in descending order of the nine essential amino acids (g/100 g protein), whereas buckwheat, rice, amaranth, corn, quinoa, and wheat are in descending order of the nine non-essential amino acids. Based on these comparisons, each grain has a unique amino acid composition. However, more research is needed on cereals, particularly pseudo cereals, which are a current trend in human diets because they are gluten-free (GF) grains with excellent nutritional and nutraceutical properties [68,84,85,86], to confirm the contents of essential and non-essential amino acids in protein of various whole grains.

## 4. Dietary Fibers and Phenolic Acids in Pseudo Cereals and Cereals, and Their Antioxidant Properties

**Figure 2:**
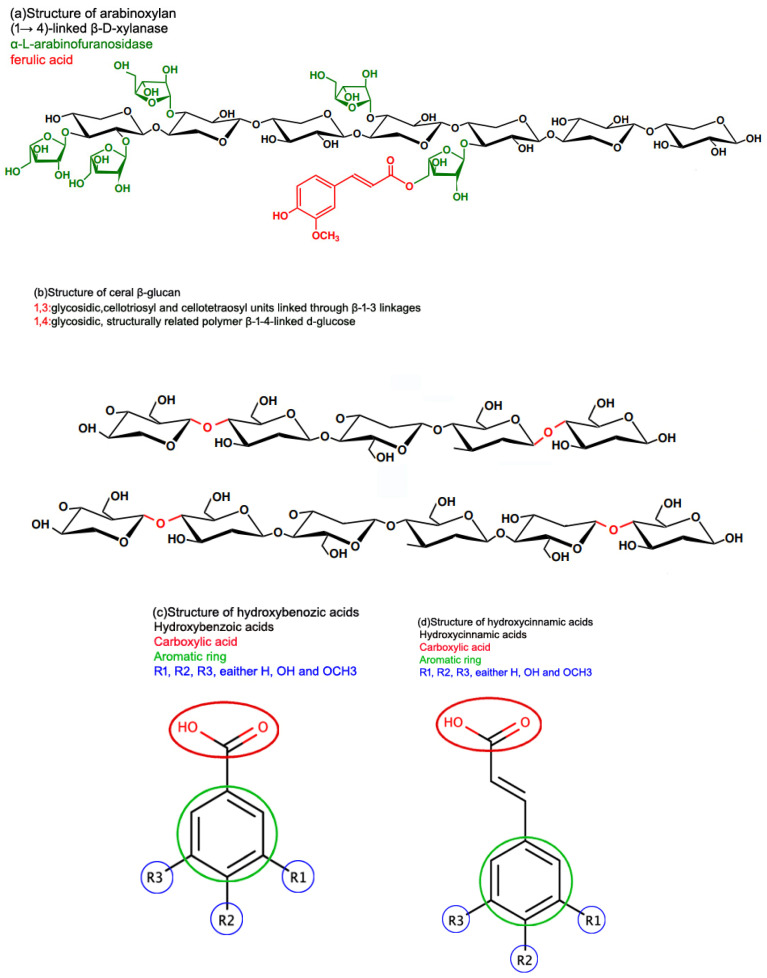
General structure of dietary fibers and phenolic acids (**a**) arabinoxylan molecular structure, (**b**) β-glucan, (**c**) hydroxybenzoic, and (**d**) hydroxycinnamic structures. R1, R2, and R3 represent H, OH, and OCH3.

Dietary fiber is defined by the European Food Safety Authority [99] as “non-digestible carbohydrates plus lignin, including non-starch polysaccharides”. Functional fiber is made up of isolated, non-digestible carbohydrates that have physiological benefits to humans [100]. Dietary fibers are divided into two categories depending on their water solubility: soluble (e.g., β-glucan) and insoluble (e.g., arabinoxylan AX) [101]. AX molecules consist of a linear backbone of D-xylopyranosyl residues connected by β-(1-4) glycosidic interactions and α-L-arabinofuranosyl residues can be linked to D-xylopyranosyl residues at O-2 and O-3 locations (Figure 2). AX has four structural elements: non-substituted, O-2 or O-3 monosubstituted, and disubstituted D-xylopyranosyl [102]. Ferulic acid may be esterified with arabinose residues at the O-5 position [103]. These ferulic acid structures can build bridges between AX chains, increasing the AX molecular weight while decreasing its water extractability. The presence of ester-linked hydroxycinnamic acids distinguishes graminaceous AXs. Trans-ferulic acid is the most abundant phenolic acid generated during alkaline hydrolysis of cereal grain cell wall material, present in considerably smaller amounts with cis-ferulic, sinapic, and trans and cis-p-coumaric acids [104]. Ferulates and diferulates also interact with lignin, forming AX-lignin cross-links. However, on a molar basis, more than half of the ferulates in grains are monomeric and do not form dimers [105].

Amaranth, quinoa, and buckwheat are pseudo cereals with a long history of use as food sources having extremely interesting nutritional properties [106]. Pseudo cereals have grown in popularity as a component in gluten-free goods during the last decade. Although dietary fiber amount varies across quinoa species, quinoa contains around 7 to 26% TDF [35,61,66,107,108], whereas amaranth’s total dietary fiber content varies between 9 and 21% (dry weight basis) [35,50,107,109,110]. Of the pseudo cereal grains, buckwheat has the lowest amount of dietary fiber [33,35,51,111] (Table 3). Quinoa and amaranth contain a higher amount of dietary fiber (~22–26%) than wheat (about 17%) [112,113], rice (about 10%) [114,115], and corn (about 19%) [116,117]. In comparison to amaranth and quinoa, buckwheat groats have a lower total dietary fiber content (7–19.0%) [33,35,51,111]; the water-soluble fiber in buckwheat seeds is mostly classified as pectin, arabinogalactan, and xyloglucan [118]. Moreover, pectin is found in the outer and inner epidermis’ cell walls as well as the endosperm of buckwheat seeds [118].

Phenolic acids are a type of secondary metabolite that belongs to a larger group of phenolic compounds found throughout the plant kingdom. They have been considered essential dietary ingredients contributing to flavor, color, and nutritional value. Phenolic compounds are distinguished by the presence of one or more aromatic rings linked by one or more hydroxyl groups. Benzoic acid and cinnamic acid derivatives are examples of phenolic acids. In general, “phenolic acids” refer to phenols with a single carboxylic acid activity. These naturally occurring phenolic acids have two distinct carbon structures: hydroxycinnamic and hydroxybenzoic. Although the fundamental structure stays constant, the number and location of hydroxyl groups on the aromatic ring dictate the diversity. Hydroxycinnamic acids are a group of aromatic carboxylic acid with a C6-C3 structure, whereas hydroxybenzoic acids are C6-C1 (Figure 2).

In Table 3, we reviewed the phenolic acids in targeted cereal grains, indicating that cereal grains have a higher amount of hydroxycinnamic than pseudo cereal grains. On the other hand, pseudo cereal grains have the highest amount of hydroxybenzoic acids compared to cereal grains. Quinoa grain contains the highest phenolic acid level with 1672–3083 µg/g dry weight [119], whereas buckwheat has the lowest amount containing 49.9 µg/g [120] followed by corn with 601–1740 µg/g [121,122]. Wheat has 658–1171 µg/g [123], amaranth has 212–570 µg/g [50,51], and rice has 300–360 µg/g [121].

Hydroxycinnamic and hydroxybenzoic acids are phytochemicals found in certain foods. It should be highlighted that in humans, circulating hydroxybenzoic acids can be the result of bacterially mediated polyphenol metabolism in the lower intestine [124,125]. Whole grain cereals and pseudo cereals have differing amounts of hydroxycinnamic and hydroxybenzoic acids. According to Table 2, the highest content of hydroxycinnamic acids is found in corn (5.7–1387.5 µg/g) [126,127], while the lowest content is found in amaranth (1.6–55.4 µg/g) [128], followed by rice (1.0–301.7 µg/g) [121,129], wheat (3.4–195.0 µg/g) [129,130], quinoa (7.0 to 150.0 µg/g) [131,132], and buckwheat (1.7–122.8 µg/g) [120,133], respectively. Moreover, wheat contains the highest content of hydroxybenzoic acids (7.5 to 230.6 µg/g) [129,130] and corn the lowest (0.5–116.5 µg/g) [126,127] followed by quinoa (13.8–110.0 µg/g) [132], amaranth (1.8–173.5 µg/g) [128], wheat (1.2–118.0 µg/g) [120,133], and rice, which ranges from 2.8 to 115.6 µg/g [121,129,134].

**Table 3:** Dietary fibers, TDF; total dietary fibers, IDF; insoluble dietary fibers, SDF; soluble dietary fibers (g/100 g), PA; phenolic acids, HC; hydroxycinnamic and HB; hydroxybenzoic acids (µg/g), contents of pseudo cereals and cereals.

Pseudo Cereals							
Whole Grains	TDF	IDF	SDF	PA	HC	HB	References
Quinoa	7.0–26.5	9.9–12.2	0.4–2.9	1672–3083	7.0–150.0	13.8–110.0	[35,50,61,66,107,108,131,132]
Amaranth	2.7–20.6	7.7–9.1	2.7–3.7	212–570	1.6–55.4	1.8–173.5	[35,50,51,107,109,110,127,128]
Buckwheat	10.3–19.0	2.2–5.8	4.8–6.	49.9	1.7–122.8	1.2–118.0	[33,35,51,111,120,133]
Cereals							
Wheat	9.2–17.0	7.2–14.7	1.4–2.9	658–1171	3.4–195.0	7.5–230.6	[35,61,112,113,121,129,130]
Rice	2.7–9.9	1.9–5.4	0.5–2.5	300–360	1.0–301.7	2.8–115.6	[114,115,122,124,129,134]
Corn	13.1–19.6	3.1–16.0	1.5–3.6	601–1740	5.7–1387.5	0.5–116.5	[35,116,117,121,122,126,127]

The antioxidant activities of the targeted grains are summarized in Table 4, which shows that corn contains the highest antioxidant activities among all other pseudo cereals and cereal. Cereal bran is the rich source of antioxidants activities [135]. For example, DPPH corn contains the highest antioxidant content (350.29 mmol Trolox/100 g) [71,136] followed by rice (180.41 mmol Trolox/100 g) [137,138,139], and wheat contains the lowest among all other grains (20.9 mmol Trolox/100 g) [140,141,142,143,144].

Compared to other grains, amaranth possess the highest content of ABTS and FRAP (179.8 µmolTrolox/100 g) and (147.4 µmolTrolox/100 g), respectively [145,146,147]. The content becomes variable in the rest of the grains. Overall, corn contains the highest content of antioxidant activity [136,148,149,150], followed by amaranth [135,146,151], quinoa [147,151,152,153], wheat [140,141,142,143,144], buckwheat [151,154,155,156], and rice [137,138,139,144].

**Table 4:** Antioxidant activities of pseudo cereals and cereals.

Pseudo Cereals						
Whole Grains	DPPH	FRAP	ABTS	TEAC	CUPRAC	References
Quinoa	60.22 (mmol Trolox/100 g)	58.7 (mg Trolox/100 g dw)	8.61 (µmolTrolox/100 g)	59.56 (µmolTrolox/100 g)	4.968 (µmolTrolox/g)	[147,151,152,153]
Amaranth	134.8 (mmol Trolox/100 g)	147.4 (µmolTrolox/100 g)	179.8 (µmolTrolox/100 g)	_	5.11 (µmol Trolox/g	[145,147,151]
Buckwheat	80.80 (mmol Trolox/100 g)	49.43 (µmolTrolox/100 g)	58 (mmolTrolox/100 g)	5.93 (µmol/100 g)	4.14 (µmol Trolox/g)	[151,154,155,156]
Cereals						
Wheat	20.9 (mmol Trolox/100 g)	70.0 (µmol TE/g dw)	5.4 (mmolTroex/100 g)	17.8 (mmol TEAC/g)	13.44 (mmol Trolox/g)	[140,141,142,143,144]
Rice	180.41 (mmol Trolox/100 g)	46.5 (µmolTrolox/100 g)	1.78 (mgTEAC/g extract)	21.3 (mg Trolox equiv/100 g)	3.21 (µmol Trolox/g)	[137,138,139,144]
Corn	350.29 (mmol Trolox/100 g)	30.56 (μmolTrolox/100 g)	92.69 (μmol Trolox/100 g)	59.6 (µg/mL)	22.78 (mg/g)	[136,148,149,150]

In summary, Table 3 shows that quinoa has the greatest total dietary fiber content (7 to 26%) followed by amaranth (2 to 21%), while rice has the lowest dietary fibers level among cereals (2–10%). In pseudo cereals, quinoa has the highest amount of total dietary fibers and buckwheat has the lowest, whereas corn has the highest content of total dietary fiber and rice contain the lowest. The ranking becomes variable in the rest of grains. Furthermore, in Table 3, we have summarized the phenolic acids in targeted grains, which indicate that the amount of hydroxycinnamic is more abundant in cereals grains than in pseudo cereals grains. On the other hand, pseudo cereal grains have a higher content of hydroxybenzoic acids than cereal grains. According to Table 4, amaranth has the highest level of antioxidant activity in pseudo cereals, whereas corn has the highest content among cereal grains. Overall, maize contains the highest content of antioxidant activity followed by amaranth, quinoa, wheat, buckwheat, and rice. The rank becomes variable in the remaining grains. These comparisons indicate that each grain prefers a different synthesis pathway to the others, resulting in a distinct profile of bioactive compounds in grains. Whole grains contain various nutrients, including dietary fiber, phenolic acids, and antioxidants. Table 4 shows that maize has the highest antioxidant content, whereas rice has the lowest. The germ and bran contain the majority of the bioactive components, which are reduced during the grain refining process. However, further research is needed to verify their results. Whole grain consumption has been associated to a lower risk of chronic non-communicable illnesses such as type 2 diabetes [6,7,24,25] and cancer [8,9,10,26,27], since it improves glycemic management, blood lipids, body weight, and inflammation while lowering premature mortality [102,157]. However, more study and communication on these health advantages is needed to translate the science behind these beneficial effects into useful data. Because these health advantages are interconnected, frequently synergistic, and individual-specific, it is difficult to obtain solid evidence of the health impacts associated with any bioactive component found in grain. Consumers today are attracted to value-added products and are health-conscious, so it will be beneficial for customers if supplements are added or if whole grains of high nutritional value replace the refined grains. Furthermore, public education is also necessary to encourage people to eat more whole grains at the recommended amounts.

## 5. Grains in Modern Times

**Figure 3:**
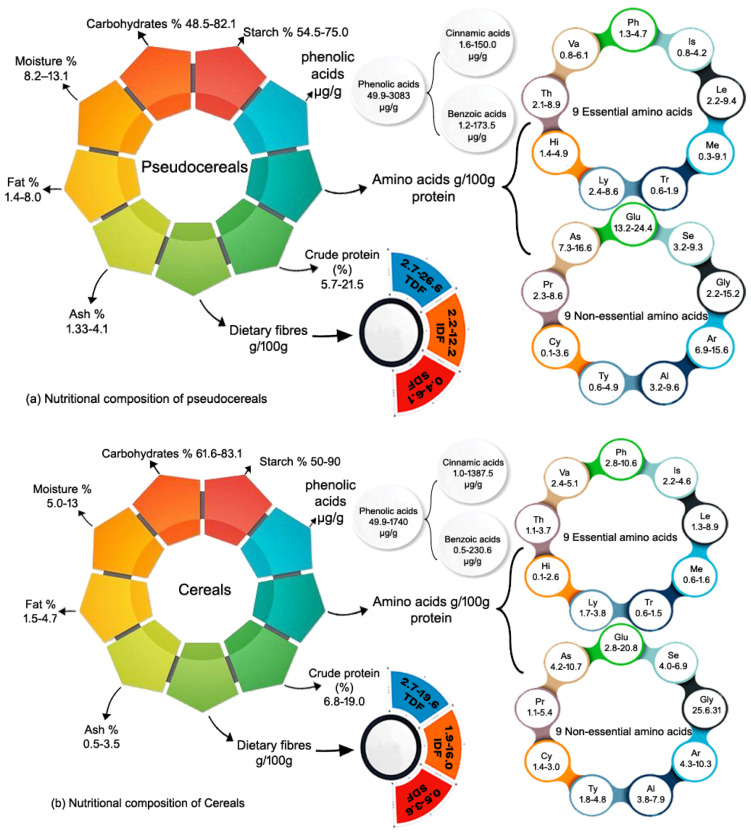
Summary of Table 1, Table 2 and Table 3 in graphical form TDF; Total dietary fibers, SDF; Soluble dietary fibers, IDF; Insoluble dietary fibers, T; Threonine, V; Valine, P; Phenylalanine, I; Isoleucine, L; Leucine, M; Methionine, T; Tryptophan, L; Lysine, A; Aspartate, G; Glutamate, S; Serine, H; Histidine, G; Glycine, A; Arginine, A; Alanine, T; Tyrosine, C; Cysteine, P; Proline.

Whole grain cereals and pseudo cereals are currently popular in human diets due to their high nutritional and nutraceutical value. As previously discussed in detail, whole grains have excellent nutritional properties and have been linked to a reduced risk of numerous chronic non-communicable diseases, including T2D [6,7,24,25] and cancer [8,9,10,26,27]. In this context, it was critical to review the nutritional and functional profile of whole grains, which possess a high nutritional profile and are now recognized as a super grain. Whole grain pseudo cereals and cereals are known as “the grains of the modern time” due to their high nutritional content [158]. Pseudo cereals such as quinoa (*Chenopodium quinoa Willd*.), amaranth (*Amaranthus* spp.), and buckwheat (*Fagopyrumesculentum Moench.*) are promising crops for the future; they are gluten-free and have high nutritional and nutraceutical value [50,51,68]. Furthermore, recent study has highlighted the potential health advantages of pseudo cereals, confirming these crops as essential resources for functional food development [50,51]. Furthermore, whole grain cereals such as wheat (*Triticum aestivum* L., *Triticum durum Desf.*), rice (*Oryza sativa* L.), and corn (*Zea mays* L.) are a major source of energy worldwide. Cereal grains and its products have a long history of use by humans. Cereals are staple foods that provide essential nutritional value in both low- and middle-income countries. Both cereals and pseudo cereals are abundant in protein and carbohydrates and they contain a good balance of essential amino acid composition that is characterized by an abundance of sulfur-rich amino acids; they are also a good source of dietary fiber and phenolic acids, with the highest amount of these compounds found in the bran and germ (Figure 3). Consumers nowadays are health conscious and are attracted towards value-added products, so the addition/incorporation of supplements or the complete replacement of refined common cereals with whole grains of higher nutritional value will benefit the consumers. There is also a need to educate the public to increase their intake of whole grains to the recommended levels.

## 6. Intake of Whole Grains and Human Health

### 6.1. Relationship between Intake of Pseudo Cereals and Cereals, and T2D

T2D is a leading cause of morbidity and mortality globally, particularly in low-income countries [159]. According to the International Diabetes Federation 2021 report, 537 million adults worldwide suffer from diabetes, with the predication of 783 million affected by 2045, indicating an increased risk in the coming decades [1]. Furthermore, according to a WHO report, worldwide, about 422 million people have T2D and 1.5 million deaths are directly related to T2D each year. The number of cases and prevalence of T2D have steadily increased over the last few decades [2]. T2D is associated with a number of lifestyle factors including diet, physical activity, obesity, alcohol, smoking, and a poor diet; among them, diet is the most important factor [160]. Whole grain pseudo cereals and cereals are essential parts of a healthy diet [161] as they contain the outer bran, germ, and inner endosperm [162], all of which are high in dietary fiber, antioxidants, and micronutrients [18], which have attracted researchers’ interest in investigating the influence of consuming whole grains on human health [163]. Intake of whole grain pseudo cereals and cereals were found beneficial for T2D [17,164,165,166,167,168,169,170,171,172,173] (Table 5). In contrast, some studies on refined grains have found a relationship between high refined grain consumption and an increased risk of T2D [174,175,176,177]. In a four-week prospective and double-blind randomized trial on T2D, the authors found that consuming 25 g of quinoa flakes significantly reduced blood triglycerides, low density lipoprotein, and total cholesterol [164]. The authors also found antioxidant abilities in rats on a high-fructose diet, decrease blood glucose, triglycerides, low density lipoprotein, and total cholesterol. Furthermore, a recent study by Khan et al. [165] found that quinoa powder can significantly reduce blood glucose levels (10–40%) in an animal model over five weeks. The goal of this research was to examine the chemical composition, total phenolic and antioxidant activity of quinoa seed powder, and its effect on diabetic rats. The author discovered that consuming quinoa at various concentrations reduced blood and glucose levels from 236.7 mg/dL to 98.7 mg/dL at 40% quinoa and 120.3 mg/dL at 30% quinoa, with an average fasting level of 64.3 mg/dL. Furthermore, rats fed quinoa seed powder had significantly decreased thyroid hormone levels (T3 and T4). Kasozi et al. [166] conducted an animal study to determine the key changes in s100a1 protein levels, as well as antioxidant and histopathologic changes in blood, renal, and hepatic tissues of male diabetic Wistar rats. This study discovered that grain amaranth supplementation increased expression of the s100a1 calcium transport proteins, which led to enhanced calcium homeostasis in tissues. The significant antioxidant activity of grain amaranth stimulated increased s100a1 protein levels, resulting in improved tissue protection against oxidative stress, which is frequent in T2D. Furthermore, [17] investigated the inhibitory activity of protein hydrolyzates derived from amaranth grain and their effect on postprandial hyperglycemia found that protein hydrolyzate can enhance glucose tolerance. Increased plasma insulin levels in both acute and chronic conditions. These studies give evidence for grain amaranth’s mechanism of action in the treatment of T2D. In another animal study, [167] found that dietary SDF supplementation decreased fasting blood glucose levels, increased oral glucose tolerance, increased liver glycogen and insulin levels, and improved serum and liver lipid profiles [168]. Furthermore, Kyrø et al., [169] investigated the relationship between whole grain intake and T2D and demonstrated that an individual who consumed 16 g of whole grains per day had an 11% and 7% decreased risk of type 2 diabetes, respectively. In this regard, [170] found that consuming black grain wheat (>69 g/d) for five weeks increased TNF-α and IL-6 levels, indicating a potential risk reduction for T2D. A higher intake of whole grains and various commonly consumed whole grain foods, such as whole grain breakfast cereal, dark bread, brown rice, added bran, and wheat germ, was substantially related to a decreased risk of T2D. These findings provide support to the present recommendations for increasing whole grain consumption as part of a healthy diet to prevent T2D [171]. On the other hand, higher consumption of white rice is linked to an increased risk of incidence of T2D. In this regard, several studies have demonstrated the association between white rice consumption and an increased risk of T2D [174,175,176,177]. Furthermore, Huang et al. [172] found that, purple corn has antidiabetic benefits by protecting pancreatic β-cells, increasing insulin production, and activating protein kinase in the liver [172]. The author suggests that it may be beneficial in the prevention of T2D and its complications. In another similar study, the consumption of maize and wheat peptide decreased serum levels and pancreatic gene expression of IL-6 and insulitis, while increasing pancreatic β-cell areas, pancreatic gene expression of IL-10, and serum levels of serine and histidine [173]. Moreover, the serum levels of serine and histidine were significantly increased in mice treated with this peptide mixture. These findings indicated that a combination of corn and wheat peptides could prevent T2D.

### 6.2. Potential Mechanism of Whole Grains and T2D

**Figure 4:**
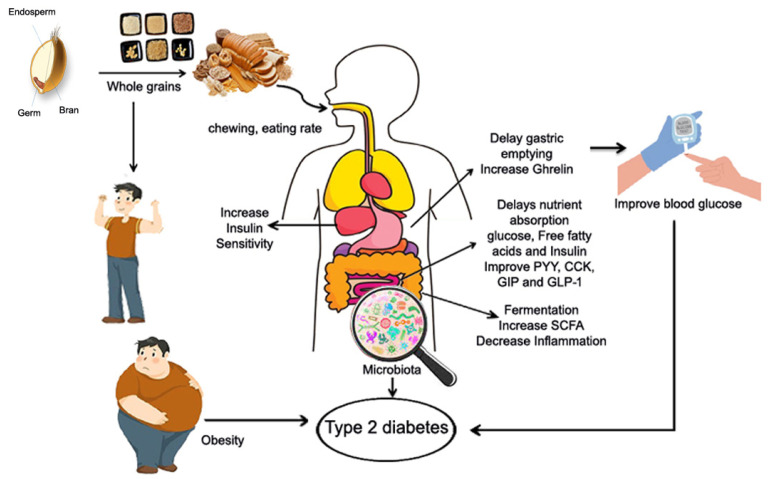
Potential mechanism of whole grains intake and reduce risk of T2D; peptide PYY; CCK: cholecystokinin; GIP: gastric inhibitory peptide; GLP-1: glucagon like peptide: SCFA, short-chain fatty acids.

Potential factors behind the relationship between whole grain consumption and a lower prevalence of T2D include their nutritional value, protein, dietary fiber, and phenolic acid. Consuming whole grains may reduce the incidence of T2D through various mechanisms. The quantity of chewing required when eating whole grains varies according to particle size and structural integrity. Increased chewing may improve satiety by increasing stomach distention and stimulating gut hormone responses. Whole grain proteins slow carbohydrate digestion and absorption, lowering postprandial glucose levels and increasing insulin sensitivity [178]. Whole grain protein has anti-inflammatory properties, which help to reduce chronic inflammation that causes insulin resistance and T2D [179,180]. Whole grain protein feeds the healthy gut bacteria, which produces short-chain fatty acids that increase glucose metabolism and insulin sensitivity. Whole grain protein promotes satiety, which leads to weight management and a lower risk of T2D [181]. Many whole grain components can help improve glucose metabolism, hence preventing T2D. Dietary fiber, in particular, has received a lot of attention since whole grains are high in fiber. Previous studies have shown that people who consume more whole grains gain less weight [182]. Dietary fibers (especially viscous fibers) can promote stomach distension, which stimulates satiety signals and increases hormones involved in body weight regulation, energy homeostasis, and glucose control. Whole grain fiber has the ability to increase gastric distension and delay intestinal transit time, thereby stimulating satiety signals and increasing hormones involved in energy homeostasis and plasma glucose control (ghrelin, peptide PYY; CCK: cholecystokinin; GIP: gastric inhibitory peptide; GLP-1: glucagon-like peptide) (Figure 4) [182]. Furthermore, fiber from whole grains inhibits nutrient absorption (glucose, free fatty acids) at the intestinal level, lowering insulin demand and stimulating fat oxidation, all of which contribute to fat storage reduction. Whole grain changes gut microbiota composition and stimulates fiber fermentation, which results in SCFA synthesis. Consuming whole grains can also help prevent T2D by improving insulin sensitivity in the body [183]. Furthermore, consuming whole grains can help improve blood glucose levels by increasing satiety signals and associated hormones. Dietary fibers can control postprandial blood glucose levels by delaying stomach emptying, increasing transit time, and glucose absorption [184]. Aside from fiber, whole grains can reduce the incidence of T2D by lowering inflammatory markers such as C-reactive protein [180]. Higher levels of specific liver enzymes, such as aspartate aminotransferase, can make the body more susceptible to T2D, although these markers can be maintained within normal limits by taking whole grains [185]. Furthermore, the bacterial gut population digests insoluble fibers and produces SCFAs, which can mediate gut hormone production, impact glucose and lipid metabolism, and contribute in the risk of T2D [24,25]. Some bioactive compounds found in whole grains may play a beneficial role in T2D [186,187]. In particular, phenolic compounds and their antioxidant and anti-inflammatory properties may help to reduce the development and progression of T2D by inhibiting oxidative stress, inflammatory cytokine transcription, and chronic low-grade inflammation [188,189], thereby improving insulin sensitivity [190]. A polyphenol-rich diet has recently been found to increase glucose tolerance and insulin sensitivity in non-diabetics while also lowering the postprandial triglyceride response [191]. Finally, bioactive substances included in whole grains may help to enhance insulin sensitivity and slow the development and progression of type 2 diabetes by reducing oxidative stress, inflammatory cytokine transcription, and subclinical inflammations.

In summary, the global incidence of T2D has become a significant risk to human health in both under developing and developed countries. Diet is the most important component in minimizing the risk and controlling the development of T2D. Epidemiological studies suggest the consumption of whole grains in lowering the risk of T2D because whole grains are rich in protein, dietary fibers, and phenolic acids. We discovered a strong inverse link between whole grain consumption and T2D. Furthermore, quinoa seeds have significant benefits over other crops in terms of human nutrition and health maintenance. Quinoa seeds should be recommended for commercial use in everyday routines since they have the potential to provide additional protection against T2D. Moreover, grain amaranth supplementation boosted the calcium content of the food and improved calcium signaling in diabetic rats’ blood, kidneys, and liver. This study provides evidence for the mechanism of action of grain amaranth in the treatment of T2D, as it is widely utilized in various underdeveloped countries. More research, however, is needed to determine the role of other components found in amaranth, as this would provide more comprehensive information of the synergistic and antagonistic roles performed by diverse pathway mechanisms. Furthermore, the authors emphasized the calcium concentration in grain amaranth. Nevertheless, the effects of oxalates, exogenous calcium, and other chemical elements under feed supplementation could provide additional information that could guide prospective clinical studies. On the other hand, white rice consumption is related to a considerably increased risk of type 2 diabetes [174,175,176,177] and a higher intake of brown rice consumption is significantly associated with a decreased risk of type 2 diabetes. The findings of several studies support the current recommendations to increase whole grain consumption as part of a healthy diet for the prevention of type 2 diabetes [17,164,165,166,167,168,169,170,171,172,173]. Furthermore, whole grains’ modulatory effects on T2D are likely regulated by a variety of pathways, including the effects of protein, dietary fiber, and phenolic acids. This represents a significant research gap that must be filled by well-designed and randomized clinical studies. However, researchers have discovered largely contrasting results in this subject, emphasizing the need for additional investigation. More research on each of the impacts of bran, protein, dietary fiber, and phenolic acids on T2D is needed to fill the gap in the link between whole grains and T2D.

### 6.3. Relationship between Intake of Pseudo Cereals and Cereals, and Cancer

Cancer is the second leading cause of morbidity and mortality and is a major public health problem in the modern time. According to the WHO Cancer report 2023, there were an estimated 20 million new cancer cases and 10 million deaths worldwide in the last few years [3]. WHO have predicated, over the next two decades, the cancer burden will increase by around 60%, putting additional strain on health-care systems, individuals, and communities. The global burden is expected to rise to around 30 million new cancer cases by 2040, with the greatest increases occurring in under developed and developed countries. Consumption of whole grain cereals has been found to be associated with a lower risk of multiple types of cancer in numerous studies, including colon, colorectal, prostrate, and other types [16,18,192,193,194,195,196,197,198,199,200,201,202] (Table 6). In contrast, some research on refined grains has found a relationship between high refined grain consumption and a higher risk of cancer [203,204]. These findings complement health recommendations to replace refined grains with whole grains. In a randomized and double-blind trial control study conducted by [192], quinoa has been found to reduce cholesterol, indicating that postmenopausal women who consumed 25 g of quinoa flakes daily, rather than maize flakes, had reduced total cholesterol and LDL but higher glutathione levels [193]. Furthermore Vilcacundo et al. [194] studied the anticancer activity of quinoa peptides, demonstrating that peptides released from quinoa seeds were shown to have the ability to inhibit tumor growth in a variety of colon cancer cell lines, including Caco-2, HT-29, and HTC-116, as well as the capacity to scavenge free radicals [194]. Additionally, this study indicated that the anticancer effects were because of suppression of α-amylase, α-glucosidase, and dipeptidyl peptidase IV by inhibition of enzymes. Moreover, in a recent study by Guo et al. [205], they indicated that quinoa peptides produced by in vitro digestion had ACE inhibitory activity, which had antihypertensive effects on rats. Peptides from raw and heat-treated amaranth seeds have also shown anticancer activity by apoptosis and cell growth suppression, using HT-29 colon cancer cells and breast cancer cell lines [206]. In another study, House et al. [16] found that amaranth tricolor contains compounds that suppress tumor cell growth, also amaranth seed proteins contain peptides associated with the reduction risk of cancer. In this regard, [80] investigated the effect of amaranth on human breast cells, suggesting that amaranth may be an excellent source of bioactive peptides with antioxidant activity and intriguing anticancer properties. It was noted that buckwheat polysaccharides were found to reduce the amplification of human PC-3 prostate cancer cells rather than directly affecting their proliferation and increasing the secretion of anti-inflammatory biomarkers [194]. Additionally, Hiroyuki Tomotake et al. [195] investigated the physiologic features of high protein buckwheat flour by measuring its effects on blood cholesterol and body fat in rats and cholesterol gallstone formation in mice. This study discovered that buckwheat protein significantly lowers the cholesterol level in the liver, reducing the risk of colon cancer. In a human study of 1372 colorectal cancer cases by Kyro et al. [196], they discovered that high intake of whole grain phenolic acids were associated with a decreased incidence of colon cancer but not with total colorectal cancer, proximal colon cancer, or rectal cancer. Furthermore, in an epidemiological study of Buescher et al. [197], they demonstrated that whole grain consumption is associated with decreased colon cancer risk. This study investigated the impact of whole and refined wheat diets on colon carcinogenesis and related risk factors in an animal model and found that whole grains had a significant effect on precancerous lesions and aberrant crypt foci, while refined grains had no effect. To date, several in vivo investigations have shown that phytochemicals can suppress cancers of the gastrointestinal tract by modulating oxidative stress. For example, in a rat model of hepato-carcinogenesis, phytic acid was demonstrated to boost glutathione S-t\zzransferases activity and reduce lipid peroxidation, and these benefits were coupled with a reduction in the emergence of hepato-carcinogenesis markers [198]. This shows that phytic acid may inhibit liver tumor development by reducing oxidative stress. In another study, phytic acid was shown to boost glutathione S-transferases activity, resulting in a reduction in the frequency of colon tumors in a rat model [199]. Furthermore, supplementation with hydoxycinnamic acid, an active rice bran phenolic acid, protects colonic tissues from colon carcinogenesis produced by the procarcinogen in an animal model. This study found that hydoxycinnamic acid had significant chemopreventive efficacy against 1,2-dimethylhydrazine-induced colon cancer [200]. Reynoso-Camacho et al. [201] investigated the preventive impact of corn tortillas against colon cancer development. The study found that eating tortillas, particularly those made from white and blue maize, significantly reduced the risk of adenocarcinoma by up to 77.5%. In an animal study of 36 male transgenic rats for eight weeks, Long et al. [201] found that purple corn reduced the incidence of adenocarcinoma in the lateral prostate and inhibited the progression of prostate cancer. The authors discovered that purple corn inhibited the expression of Cyclin D1 and downregulated the activation of p38 MAPK and Erk1/2. Since purple corn color is a mixture, determining its active component should help in the understanding and usage of purple corn color for prostate cancer chemoprevention. The results suggested that cyanidin-3-glucoside and pelargonidin-3-glucoside are the active compounds in corn.

### 6.4. Potential Mechanism of Whole Grains and Cancer

**Figure 5:**
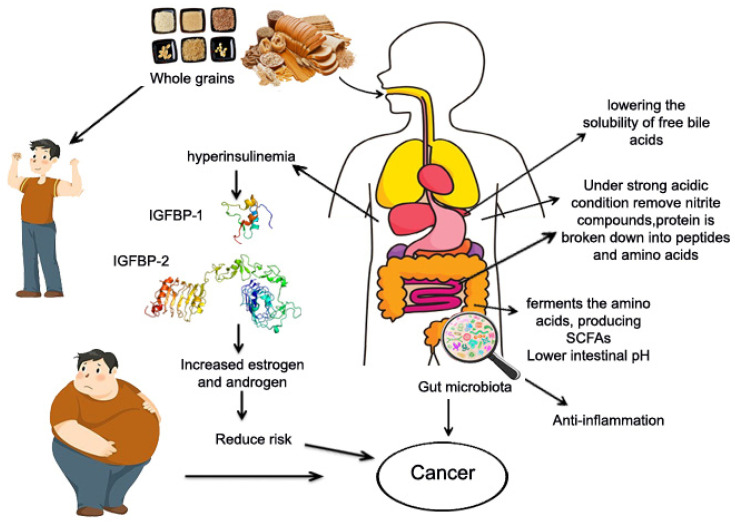
Potential mechanism of whole grains intake to reduce risk of cancer in insulin-like growth factor-binding protein 1 (IGFBP-1) and insulin-like growth factor-binding protein 2 (IGFBP-2).

Intake of whole grains has been shown to lower the risk of obesity and improve metabolic problems, as well as lower the risk of cancer [25,53]. Obesity, particularly central/visceral obesity, causes insulin resistance and prolonged compensatory hyperinsulinemia, which has been shown to induce mitogenic effects and lead to cancer risk by activating both the insulin receptor and the insulin-like growth factor binding protein 1 and 2 receptor (Figure 5); this then increased estrogen and androgen levels which have also been linked to the development of cancer. Secondly, the stomach and small intestine digest whole grain protein into peptides and amino acids [20]. Amino acids enter the bloodstream and travel to the colon (large intestine) [21]. The gut microbiota in the colon ferments amino acids, resulting in SCFAs and other metabolites [22]. Consuming whole grain protein may alter the composition of the gut microbiota, increasing the abundance of beneficial bacteria while decreasing the abundance of harmful bacteria, which inhibit cancer cell growth and proliferation, induce apoptosis, and reduces inflammation and oxidative stress [20]. Third, whole grains contain a variety of phytochemicals, which have been linked to cancer prevention [207]. Fourth, whole grains provide a good source of dietary fibers which dilute carcinogens and reduce their absorption in the intestinal epithelium by increasing feces production and shortening the intestine’s transit time. Dietary fiber can be fermented in the colon to produce SCFAs such as butyrate. Butyrate is the preferred fuel for mucosal cells, and it can stimulate apoptosis and antitumor activity, slowing tumor growth. They also lower the pH of the intestine, which reduces the solubility of free bile acids and thus their carcinogenicity.

Furthermore, dietary fiber can remove nitrite from the stomach and reduce the levels of nitroso compounds under high acid conditions. Nitrates increase the risk of stomach cancer [26]. Moreover, whole grains have antioxidant and anti-inflammatory properties, which can improve blood sugar response and reduce insulin resistance, lowering the risk of cancer [208,209]. Whole grains are high in antioxidants and phenolic acids, which can help prevent oxidative damage [186]. Whole grain foods contain high levels of bioactive compounds with anticarcinogenic properties [210]. The combined anticarcinogenic effects of these bioactive compounds are likely to explain many of the findings from these studies.

In summary, several epidemiological studies have discovered an inverse relationship between whole grain consumption and various types of cancer; longitudinal studies are needed to investigate cancer development and progression. Whole grains are a complex food matrix containing a variety of bioactive compounds that function together to prevent cancer. It is thus difficult to determine which constituent is responsible for protection; several studies suggestion the beneficial effect of whole grain dietary fiber together with phenolic acids may have beneficial effect on the reduction of cancer. For example, some whole grains’ positive effects are primarily dependent on protein, dietary fiber, and phenolic acids, but the content varies by grain and is also found in other foods consumed by high whole users. Although refined grains have a low total dietary fiber content, refining removes proportionally more insoluble fiber than soluble fiber. Challenges appear in a diverse area, so it is critical to determine which of these components will provide the most protection. More research is needed to fully understand their effects, whether bran, germ, protein, dietary fibers, or specific phenolic acids are linked to a lower risk of cancer. Furthermore, the link between the consumption of whole grains and other types of cancers, such as breast, pancreatic, oral, and pharyngeal cancer, is less studied, and the results are often conflicting. The exact mechanism and independent effect of whole grain bran, protein, phenolic acids, and dietary fibers should be investigated.

## 7. Conclusions

Whole grain pseudo cereals and cereals are known as “the grains of a modern time” due to their high nutritional and bioactive components. In terms of protein content, amaranth contains the highest content of protein and the lowest is found in rice, followed by wheat, quinoa, buckwheat corn, indicating that pseudo cereals contain the highest content of protein in comparison to cereals. Corn, quinoa, rice, buckwheat, wheat, and amaranth, are in descending order of the nine essential amino acids (g/100 g protein) while buckwheat, rice, amaranth, corn, quinoa, and wheat are in descending order of the nine non-essential amino acids (g/100 g protein). In pseudo cereals, quinoa possesses the highest content of total dietary fibers and phenolic acids, while buckwheat has the lowest. In cereal grains, corn contains the highest content of total dietary fibers and rice contains the lowest, while wheat contains the highest content of phenolic acids and rice contains the lowest. The ranks become variable in the remaining cereals. Additionally, we discovered that cereal grains contain greater amounts of hydroxycinnamic acid than pseudo cereal grains. These compounds are considered to be primarily responsible for the beneficial effects on human health. Several epidemiological studies support the consumption whole grains over refined grains. Since, whole grains improve blood lipids, body weight, inflammation, and glycemic control while lowering premature mortality, we have found a strong inverse relationship between whole grain consumption and decreased risk of T2D and cancer. Additionally, the modulatory effects of whole grains on cancer and T2D might be contributed via a number of mechanisms, such as the actions of phenolic acids, dietary fibers, and protein. Future studies should carry out randomized controlled trials to validate observational results and establish causality. In addition, the current manuscript encourages the investigation of the specific mechanisms by which whole grains exert their beneficial effects on health by examining the effects of different types of specific protein, dietary fibers, and phenolic acids that might help to prevent or treat T2D and cancer.

## Figures and Tables

**Table 5:** Summary of whole grains intake and reduction risk of T2D, ↓; decrease, ↑; increase.

Pseudo Cereals					
Whole Grains	Action Mechanism	Model	Doses	Key Findings	References
Quinoa	Antidiabetic	24 rats; 5 weeks	quinoa seeds in high-fructose diet	↓ Blood glucose, ↓ blood triglycerides, ↓ low density lipoprotein, ↓ total cholesterol	[164]
	Antidiabetic	55 male albino rats; 6 weeks	quinoa seeds powder	↓ glucose levels, ↓ thyroid hormones	[165]
Amaranth	Antidiabetic	30 rats; 5 weeks	Amaranth grains	↑ Calcium in the diet and improved calcium signaling in blood, kidney, and liver of diabetic rats. ↑ expression of the s100a1 calcium transport proteins	[166]
	Antidiabetic	25 CDI mice; 4 weeks	Amaranth protein hydrolyzate	↑ plasma insulin	[17]
Buckwheat	Antidiabetic	165 Diabetic Individuals; 4 weeks	Buckwheat	↓ level of serum glucose, ↓ fast insulin, ↓ total cholesterol, ↓ LDL cholesterol	[167]
	Antidiabetic	50 C57BL/6 mice; weeks	Buckwheat soluble dietary fibers	↓ levels of fasting blood glucose, ↑ oral glucose tolerance, ↑ levels of liver glycogen and insulin, ↑ lipid profiles in both the serum and liver.	[168]
Cereals					
Wheat	Antidiabetic	57,053 men and women aged 50–65	Whole grain Wheat	16 g of whole wheat per day lower risk of type 2 diabetes and high whole grain intake may have more benefits	[169]
	Antidiabetic	120 patients of T2D	Black wheat grains intake (>69 g/d) for 5 weeks	↑ glycemia and the inflammatory profile in T2D patients, ↓ glycated albumin, and prevented the increase in TNF-α and IL-6 levels	[170]
Rice	Antidiabetic	4,618,796 men and women	Brown rice	higher consumption of total whole grains and the most commonly consumed whole grain foods was significantly associated with a lower risk of type 2 diabetes	[171]
	diabatic	45,411 male and female aged 45–74, 25,666 men 33,622 women age 45–75 y, 132,373 individuals age 35–70 y, 13,284 cases, 2,352,384 participants	White rice	intake of white rice is associated with an increased risk of type 2 diabetes	[174,175,176,177]
Corn	Antidiabetic	4 groups mice; 8 week	Purple corn	↑ insulin secretion ↑ AMPK activation in the liver, ↑ phosphorylation of activated protein kinase, ↓ phosphoenolpyruvate carboxykinase, ↓ glucose 6-phosphatase	[172]
	Antidiabetic	6 mice; 1–6 week	corn	↓ serum level ↓ pancreatic gene expression of IL-6 and insulitis, ↑ pancreatic β-cell areas, pancreatic gene, ↑ expression of IL-10 serum levels of serine and histidine	[173]

**Table 6:** Summary of whole grains intake and reduction risk of cancer, ↓; decrease, ↑; increase.

Pseudo Cereals					
Whole Grains	Action Mechanism	Model	Doses	Key Findings	References
Quinoa	Cancer	35 females; 2 years	quinoa flakes daily	↓ interleukin-6, which is a marker of inflammation, ↓ Tumor, ↓ total cholesterol↓ serum triglyceride	[192]
	Colon cancer	In vitro gastrointestinadigestion model	Quinoa protein	Large peptides responsible for the colon cancer cell viability inhibitory activitySmaller peptides < 5 kDa with antiproliferative activity i and was the main responsible for the radical scavenging activity while peptides > 5 kDa showed greater anticancer effects.	[193]
Amaranth	Cancer	Animal model	Amaranth protein and polyphenols	Inhibitory effect on tumor cell proliferation inhibition of histone acetylation	[16]
	Breast cancer	Human breast cells	Amaranth	The results indicated that the digested sample was capable of inhibiting cell growth and found that amaranth may be a good source of bioactive peptides with good antioxidant activity and promising anticancer activity.	[80]
Buckwheat	Prostate cancer	10 weeks old mice	crude polysaccharides buckwheat	negative correlation between PC-3 cell viabilities and (interleukin [IL]-6 + tumor necrosis factor [TNF]-α)/IL-10 level ratios in the corresponding MCM, implying that macrophages suppress PC-3 cell growth through decreasing secretion ratios of proinflammatory/anti-inflammatory cytokines in a tumor microenvironment.	[194]
	Colon cancer	Mice weight 70 g and 28 g	High protein buckwheat flour	Strong activities against cancer, buckwheat protein significantly inhibited the growth of an artificially induced tumor	[195]
Cereals					
Wheat	Colorectal cancer	Human study 1372 colorectal cancer cases	Wheat phenolic acids	High concentrations of phenolic acids were associated with a lower incidence of distal colon cancer but not with overall colorectal cancer, proximal colon cancer, and rectal cancer.	[196]
	Colon cancer	Mice	Whole wheat vs. refined wheat	Red wheat had significantly fewer colonic precancerous lesions than soft white-fed, while refined grains had no reduction risk. Oxygen radical and fecal bile acid concentration were higher than refined grains	[197]
Rice	Cancer	Rat model of liver cancer	Rice bran	↑ glutathione-S-transferase activity, ↓ lipid peroxidation ↓ level of placental glutathione-S-transferase -positive foci, a marker of hepato-carcinogenesis, ↓ number of colon tumors	[198,199]
	Colorectal cancer	Rat model of colorectal cancer	Rice bran phytochemicals	Reverse the effect of chemically-induced colorectal cancer in rats, by reducing level of lipid peroxidation and protein oxidation in liver, ↑ activity of superoxide dismutase, catalase and glutathione peroxidase↑ glutathione, vitamin E and vitamin C levels↓ number of aberrant crypt foci and colon tumors	[200]
Corn	Cancer	4–5 wk, male rats	corns	inhibition of β-glucuronidase activity, and induction of detoxifying enzymes in liver and colon, as well as a decrease in the expression of the two most important proliferative proteins (K-ras and β-catenin) involved in colon carcinogenesis	[201]
	prostate cancer	36 rats; 8 weeks	Purple corn	↓ incidence of adenocarcinoma in the lateral prostate and slowed down the progression of prostate cancer.↓ expression cyclin-dependent kinases, downregulation of the activation growth factor and cytokines	[202]

## Data Availability

No new data were created or analyzed in this study. Data sharing is not applicable to this article.
